# Evaluation of Ultrasound, Microwave, Ultrasound–Microwave, Hydrothermal and High Pressure Assisted Extraction Technologies for the Recovery of Phytochemicals and Antioxidants from Brown Macroalgae

**DOI:** 10.3390/md19060309

**Published:** 2021-05-27

**Authors:** Marco Garcia-Vaquero, Rajeev Ravindran, Orla Walsh, John O’Doherty, Amit K. Jaiswal, Brijesh K. Tiwari, Gaurav Rajauria

**Affiliations:** 1 School Agriculture and Food Science, University College Dublin, Dublin D04 V1W8, Belfield, Ireland; marco.garciavaquero@ucd.ie (M.G.-V.); john.vodoherty@ucd.ie (J.O.); 2 Department of Biological & Pharmaceutical Sciences, Munster Technological University, Kerry Campus, Clash V92 CX88 Tralee, Co. Kerry, Ireland; rajeev.ravindran@staff.ittralee.ie; 3 School of Food Science and Environmental Health, College of Sciences and Health, Technological University Dublin, City Campus, Central Quad, Dublin D07 ADY7, Grangegorman, Ireland; C14429608@mytudublin.ie (O.W.); amit.jaiswal@tudublin.ie (A.K.J.); 4 Teagasc Food Research Centre, Dublin D15 DY05, Ashtown, Ireland; brijesh.tiwari@teagasc.ie

**Keywords:** seaweed, innovative technology, extraction, polyphenol, nutraceuticals

## Abstract

This study aims to explore novel extraction technologies (ultrasound-assisted extraction (UAE), microwave-assisted extraction (MAE), ultrasound–microwave-assisted extraction (UMAE), hydrothermal-assisted extraction (HAE) and high-pressure-assisted extraction (HPAE)) and extraction time post-treatment (0 and 24 h) for the recovery of phytochemicals and associated antioxidant properties from *Fucus vesiculosus* and *Pelvetia canaliculata*. When using fixed extraction conditions (solvent: 50% ethanol; extraction time: 10 min; algae/solvent ratio: 1/10) for all the novel technologies, UAE generated extracts with the highest phytochemical contents from both macroalgae. The highest yields of compounds extracted from *F. vesiculosus* using UAE were: total phenolic content (445.0 ± 4.6 mg gallic acid equivalents/g), total phlorotannin content (362.9 ± 3.7 mg phloroglucinol equivalents/g), total flavonoid content (286.3 ± 7.8 mg quercetin equivalents/g) and total tannin content (189.1 ± 4.4 mg catechin equivalents/g). In the case of the antioxidant activities, the highest DPPH activities were achieved by UAE and UMAE from both macroalgae, while no clear pattern was recorded in the case of FRAP activities. The highest DPPH scavenging activities (112.5 ± 0.7 mg trolox equivalents/g) and FRAP activities (284.8 ± 2.2 mg trolox equivalents/g) were achieved from *F. vesiculosus*. Following the extraction treatment, an additional storage post-extraction (24 h) did not improve the yields of phytochemicals or antioxidant properties of the extracts.

## 1. Introduction

Macroalgae are a diverse group of organisms with over 19,000 species identified worldwide [1], and only 5% of them are currently exploited at an industrial level for food applications [2]. Globally, the macroalgal sector was valued at USD 10.31 billion in 2018, and at an annual growth rate of 8.9% this market is predicted to be valued at USD 22.13 billion by 2024 [3]. This valuable biomass is mainly exploited as food (85%), while the remaining 15% is exploited for the generation of high value compounds with multiple applications as pharmaceuticals, nutraceuticals and cosmeceuticals that surpass in economic value any other use or application currently being considered for macroalgae [4].

The increased economic value and research interest in macroalgal compounds as pharmaceuticals, nutraceuticals and cosmeceuticals relies on the unique and wide variety of health benefits associated with their consumption. Macroalgae are able to adapt rapidly to abiotic and biotic stressors of the marine environment by producing secondary metabolites with unique chemical structures [5,6]. Thereby, abiotic stress (i.e., changes in water level, solar radiation and/or temperature) may lead to an increased production of reactive oxygen species and free radical species that may damage the structure and metabolism of algae in the long term [5,7]. As a defence mechanism, the stressed macroalgae produce high amounts of antioxidant compounds, such as polyphenols [5,6]. Polyphenols encompass a wide variety of antioxidant compounds and chemical classes that can be quantified in the biomass as total phenolic content (TPC), total flavonoid content (TFC), total tannin content (TTC), and in the case of brown macroalgae, total phlorotannin content (TPhC) [8]. These compounds have strong antioxidant properties, playing a major role in the prevention or treatment of several diseases or disorders, including obesity, diabetes, cancer and cardiovascular diseases, amongst others [5,6]. Brown macroalgae *F. vesiculosus* and *P. canaliculata* tested in this study are native species of Ireland which have reported a high amount of polyphenols and polysaccharides with potential health properties [9,10]. Moreover, Chater et al. [11] reported that ethanol extracts of selected seaweeds showed significant inhibition of lipase in a model gut system, suggesting their potential in weight management.

Polyphenols from macroalgae are typically extracted using conventional technologies, by macerating the biomass in organic solvents (methanol, ethanol, and acetone) at room temperature or heating the mixtures for several hours or days [12]. Further processing of the polyphenol extracts is also required at a later stage, aiming to eliminate carbohydrates that are normally tightly bound to these compounds using one or several purification processes [13]. The exploitation of TPC, TFC, TTC and TPhC at an industrial level requires processing the biomass to extract high yields of these compounds with low or minimal damage to their antioxidant properties, while minimising the co-extraction of carbohydrates or total sugar contents (TSC). Novel technologies, mainly UAE, MAE, UMAE, HAE and HPAE, and natural deep eutectic solvents have gained momentum as more efficient and environmentally friendly procedures for the extraction of multiple compounds from macroalgae, as seen in the recent scientific literature [8,14,15,16,17]. Novel extraction technologies and protocols are regularly described as being efficient, achieving high yields of targeted compounds, while minimising energy consumption, the time of extraction and the need for organic solvents, thus improving the sustainability of these procedures as well as the safety of the operators during these extraction processes [16,18,19]. Most of the literature available on polyphenols from macroalgae focuses on the chemical characterisation of the polyphenols extracted [20,21,22], on using novel extraction technologies to optimise the yields of compounds extracted [12,23,24], or comparing the efficiency of novel technologies against conventional protocols [25]. However, limited studies are available comparing the efficiency of several novel extraction technologies for the extraction of polyphenols and antioxidants from brown macroalgae [14].

The overarching objective of the study was to identify the best novel extraction technology (while keeping solvent type, concentration and extraction time constant) to recover polyphenols with high antioxidant properties from macroalgal biomass. To achieve this, the study aimed to explore (1) multiple novel extraction technologies (UAE, MAE, UMAE, HAE and HPAE) using fixed extraction conditions (solvent: 50% ethanol; extraction time: 10 min; algae/solvent ratio: 1/10) and (2) the effect of extraction time post-treatment (0 and 24 h) on the extraction of polyphenols (TPC, TFC, TTC and TPhC) and carbohydrates (TSC), as well as the antioxidant properties (DPPH and FRAP) of the extracts generated from brown macroalgae *F. vesiculosus* and *P. canaliculata*.

## 2. Results and Discussion

### 2.1. Effect of Technologies on the Yields and Phytochemical Contents of Extracts

**Figure 1:**
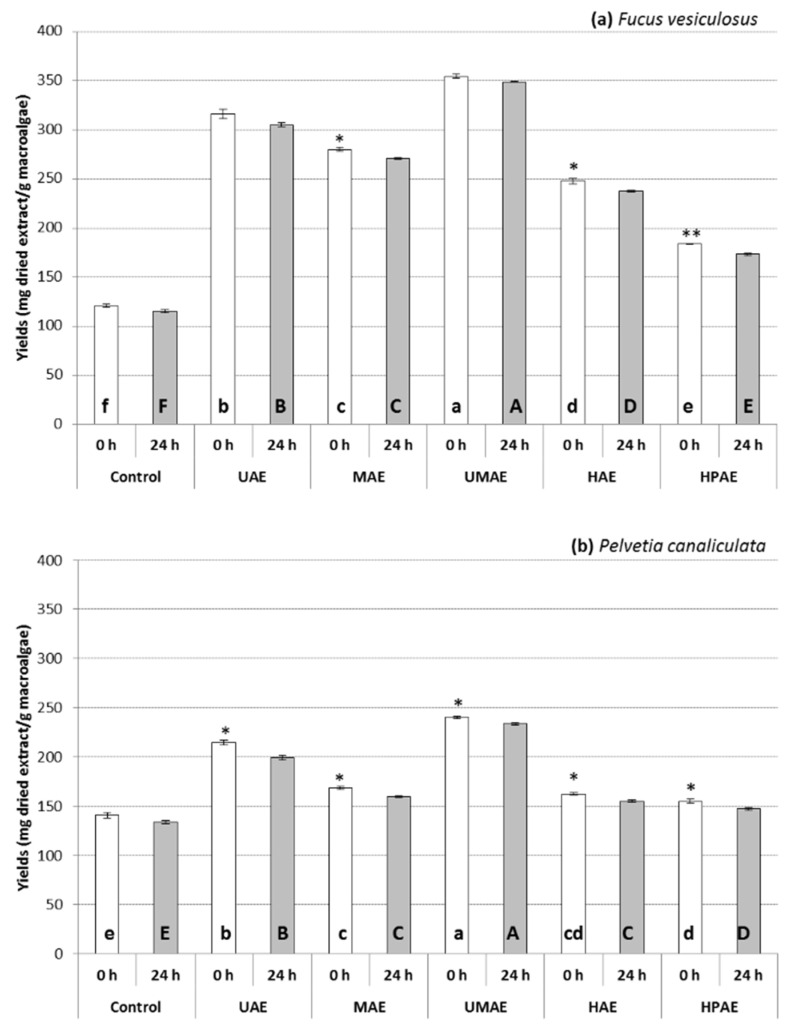
Yields of extract obtained from *F. vesiculosus* (**a**) and *P. canaliculata* (**b**) following control, ultrasound-assisted extraction (UAE), microwave-assisted extraction (MAE), ultrasound–microwave-assisted extraction (UMAE), hydrothermal-assisted extraction (HAE) and high-pressure-assisted extraction (HPAE) treatments. Results are expressed as average ± standard deviation (*n* = 4). Different letters indicate statistical differences (*p* < 0.05) on the yields of macroalgal extract between the different technological treatments incubated for 0 h (lower case letters) or 24 h (upper case letters). Statistical differences between the extraction yields following different extraction times post-treatment (0 or 24 h) within the same extraction treatment are reflected in the figure as ns (non-significant), * *p* < 0.05 and ** *p* < 0.01.

The extraction yields, expressed as mg of dried solids extracted per g of biomass by each novel extraction technology with or without additional extraction time post-treatment (0 and 24 h), are represented in Figure 1. In general, the yields of compounds extracted were higher from *F. vesiculosus* (yields ranging from 173.33 to 354.67 mg dried extract per g macroalgae) compared to *P. canaliculata* (147.00 to 240.33 mg dried extract per g macroalgae). Additionally, 24 h extraction of the samples following the technological treatments did not improve the extraction yields from both macroalgae. Overall, the yields of compounds extracted from both macroalgae were the highest using UMAE (354.67 ± 2.33 and 240.33 ± 1.20 mg dried extract per g macroalgae for *F. vesiculosus* and *P. canaliculata*, respectively), followed by UAE (316.33 ± 4.63 from *F. vesiculosus* and 214.33 ± 2.60 from *P. canaliculata*), while variable results were achieved for the other technologies explored. HPAE achieved the lowest yields from both *F. vesiculosus* (184.00 ± 0.58 mg dried extract/g macroalgae) and *P. canaliculata* (155.00 ± 2.31 mg dried extract/g macroalgae).

The effect of different extraction technologies and extraction time on the extraction of phytochemicals (TPC, TPhC, TFC, TTC and TSC) from *F. vesiculosus* and *P. canaliculata* is presented in Table 1. There was a significant variation in the extraction of each phytochemical depending on the macroalgal species, technological treatments and extraction times. In general, extracts from *F. vesiculosus* contained the highest levels of TPC, TPhC, TFC, TTC and TSC compared to *P. canaliculata*. The differences in the recovery of phytochemicals between both species could be attributed to inter-species differences; however, the effect of technological treatments on both species was similar. Garcia-Vaquero et al. [7] reported huge variation in the TPC, TSC and other relevant phytochemicals produced by the brown macroalgae *Laminaria digitata*, *Laminaria hyperborea* and *Ascophyllum nodosum* collected in Ireland every season for up to 2 years. Thus, the levels of TPC, TPhC, TFC, TTC and TSC extracted in this study may be strongly influenced by the initial concentration of these compounds in the biomass. Moreover, Harnedy and FitzGerald [26] mentioned that strong cell walls containing variable types and amounts of polysaccharides depending on the macroalgal species were one of the main obstacles hindering an efficient extraction of compounds from macroalgae. Thus, the type of polysaccharides and ionic interactions taking place with other cell wall constituents will be strongly influenced by the macroalgal species studied [27,28], affecting the yields of compounds extracted depending on the macroalgal species.

When exploring the extraction of the samples post-treatment with different novel extraction technologies, an additional extraction of the samples for 24 h did not enhance the phytochemical contents of the extracts (see Table 1). The extraction of the samples for 24 h was performed under refrigeration conditions (4 °C) to avoid any damage to the antioxidant properties of the extracts during prolonged extraction times. An additional release of compounds may have occurred during the extraction of the samples, although the overall recovery of compounds in the extracts may even decrease due to the precipitation of solubilised compounds in ethanolic solutions during the extraction process [29].

Focusing on the use of novel technologies without additional extraction time, overall, UAE achieved the highest yields of phytochemicals from both *F. vesiculosus* and *P. canaliculata*. UAE generated extracts containing the highest yields of TPC (445.0 ± 4.6 mg gallic acid equivalents (GAE)/g dried extract), TPhC (362.9 ± 3.7 mg phloroglucinol equivalents (PGE)/g dried extract), TFC (286.3 ± 7.8 mg quercetin equivalents (QE)/g dried extract), and TTC (189.1 ± 4.4 mg catechin equivalents (ChE)/g dried extract) from *F. vesiculosus*. This technology also achieved the highest yields of TPC (250.6 ± 6 mg GAE/g), TPhC (203.9 ± 4.9 mg PGE/g), TFC (122.6 ± 3.4 mg QE/g) and TTC (79.5 ± 4.6 mg ChE/g) from *P. canaliculata*. UAE has been previously used to extract polyphenols and antioxidants from brown macroalgae, although the extraction yields and antioxidant properties may vary depending on the UAE technology used, the extraction conditions and the biomass (species, season and place of collection). Vázquez-Rodríguez et al. [30] optimised UAE for the recovery of polyphenols and carbohydrates from the brown macroalgae *Silvetia compressa*. The authors reported high TPhC (10.82 mg PGE/g) using maximum ultrasound power and medium polarity solvents (62.5% ethanol solution) [30]. Ummat et al. [24] reported high yields of TPC (572.3 ± 3.2 mg GAE/g), TPhC (476.3 ± 2.2 mg PGE/g) and TFC (281.0 ± 1.7 mg QE/g) extracted from *F. vesiculosus* using UAE during 30 min and 50% ethanol as an extraction solvent. Hassan, Pham and Nguyen [23] explored UAE (60 min, 60 °C and 60% ethanol as extraction solvent) for the extraction of TPC (9.07 ± 0.49 mg GAE/g), DPPH (16.11 ± 1.69 mg TE/g) and FRAP (9.03 ± 0.58 mg TE/g) from *Padina australis*. Previous studies have reported that the algal cell wall contains variable types and amounts of complex polysaccharides depending on the type of macroalgal species, which is one of the main obstacles hindering an efficient extraction of compounds from them [26,31]. Additionally, the amount of bioactive in macroalgae is also influenced by the geographical location and harvesting season [7]. Therefore, to recover highest yield of bioactive, a specific extraction strategy focusing on extraction parameters and UAE conditions as well as harvesting season for each individual algal species is important.

MAE in the current study showed a significantly low value of TPC compared to the other novel technologies used in this study, but higher than the control. MAE exhibited 23.6% and 17.8% higher TPC than the control but up to 12% and 18% lower TPC than other technologies for *F. vesiculosus* and *P. canaliculata,* respectively. MAE in the current study did not show any advantage compared to the other novel technologies used in this study. In the case of *P. canaliculata*, it generated the extracts with the lowest levels of TFC (94 ± 8 mg QE/g) and TTC (36 ± 4 mg ChE/g), with concentrations comparable to those of the control samples (83 ± 8 mg QE/g and 34 ± 2 mg ChE/g). Amarante et al. [32], using optimised MAE conditions (75 °C, 5 min and 57% ethanol), achieved low yields of TPhC (9.8 ± 1.8 mg PGE/g extract) from *F. vesiculosus* comparable to those achieved using conventional solvent extraction (11.1 ± 1.3 mg PGE/g extract). However, Yuan et al. [12] recovered high yields of TPC from brown macroalgae (*A. nodosum*, *Laminaria japonica*, *Lessonia trabeculate* and *Lessonia nigrecens*) by MAE using a UWave-2000 reactor for microwave irradiation (2.45 GHz) at 110 °C for 15 min (5 min climbing and 10 min holding). The low extraction yields of phytochemicals in the current study may be due to operational limitations of the power of the MAE used (250 W). Similar to these results, while exploring MAE to extract phytochemicals from *A. nodosum*, the maximum yields of TPC (1790.93 ± 112.11 mg GAE/100 g biomass) were achieved by applying 600 W of microwave power for 5 min compared to treatments at 250 and 1000 W [14].

UMAE did not improve the extraction yields of phytochemicals compared to UAE in *F. vesiculosus*, while in the case of *P. canaliculata*, UMAE achieved levels comparable to those of UAE, with high yields of TPC (238 ± 3 mg GAE/g), TPhC (194 ± 2 mg PGE/g) and TFC (114 ± 3 mg QE/g). The efficiency of ultrasounds and microwave extraction forces combined seems to be influenced by the macroalgal species studied. UMAE has been previously explored for the extraction of multiple compounds from terrestrial crops, such as soluble dietary fibre from coffee silverskin [33], pectin from potato pulp [34], and lycopene from tomatoes [35], while limited literature is available on the use of UMAE in macroalgae. To our knowledge, Garcia-Vaquero et al. [14] was the first report analysing the application of UMAE to extract phytochemicals from *A. nodosum*. The authors achieved extracts with the highest contents of TSC (10409 ± 229.11 mg glucose equivalents (GlcE)/100 g biomass) and TPC (2605.89 ± 192.97 mg GAE/100 g biomass) by using UMAE compared to UAE and MAE.

Extracts generated by HAE achieved high levels of TPC (433.2 ± 6.2 mg GAE/g) and TPhC (353.3 ± 5.1 mg PGE/g) from *F. vesiculosus*, comparable to those achieved using UAE. Moreover, HAE was the most efficient method to recover TSC from *F. vesiculosus* (239.4 ± 2.9 mg GlcE/g) and *P. canaliculata* (175.7 ± 9.0 mg GlcE/g). Similar to these results, Rajauria et al. [8] reported the efficiency of HAE (85–121 °C, 15 min using 60% methanol as a solvent) to recover TPC, TPhC and TSC from brown macroalgae *Laminaria saccharina*, *L. digitata* and *Himanthalia elongata*. In the case of carbohydrates, HAE (120 °C, 80.9 min) obtained extracts containing high yields of fucose-containing polysaccharides from *L. hyperborea* (2782 mg fucoidan/100 g biomass) [36]. The efficiency of HAE to disrupt cell walls of multiple algae and increase the extraction of compounds has also been demonstrated for multiple carbohydrates, i.e., Lemus et al. [37] used HAE (121 °C, 3 h) to extract agar from red macroalgal species *Pterocladia capillacea*, *Gelidium floridanum* and *Gelidium serrulatum*, with extraction yields ranging from 31.7 to 33% of the total biomass. HAE (103 kPa, 15 min) was also used by Lee et al. [38] to extract agar from multiple *Gracilaria* spp.

The application of HPAE did not have a significant advantage when exploring the extraction of phytochemicals from both *F. vesiculosus* and *P. canaliculata*. HPAE achieved high yields of TFC (110.7 ± 5.6 mg QE/g) from *P. canaliculata* comparable to those levels achieved by UAE and UMAE. To our knowledge, there are no data on the efficiency of this method for the extraction of TFC. However, the efficiency of HPAE has been demonstrated for the extraction of other compounds from algal matrices, such as lipids from the microalgae *Chlorella saccharophila* [39]. Moreover, Li et al. [40] used a combination of high pressure homogenisation and HAE for the extraction of sulphated polysaccharides from the brown macroalga *Nemacystus decipients*, achieving yields of sulphated carbohydrates of approximately 17%.

Overall, all the tested novel technologies extracted significantly higher amounts of phytochemicals from *F. vesiculosus* and *P. canaliculata* compared to control samples (treated by maceration), except in the case of TSC. The extraction yields of TSC from *F. vesiculosus* and *P. canaliculata* achieved in the current study may be related to the type of polysaccharides (such as laminarins and fucoidans) produced by the biomass and how these compounds interact with the polarity of the solvent during the process of extraction [11,41,42]. Variable results are available in the literature in relation to the extraction of TSC using ethanolic solutions from multiple biological matrices. Zhang et al. [43] optimised the extraction of phytochemicals and antioxidant activities from *Asparagus officinalis* L root cultivars and reported maximum recovery of TPC, TFC, and TSC when using 75.23% ethanol at 51 °C, solid:liquid ratio 1:50 and 73.02 min of extraction. In the case of macroalgae, Vázquez-Rodríguez et al. [30] optimised the UAE of TPhC and TSC from the macroalgae *Silvetia compressa*. The authors achieved extracts with the highest yields of TSC using 25% ethanol, while solvents of high polarity increased the extraction of TPhC at 50 °C, 3.8 W/cL of ultrasonic power density, and 30 mL of solvent per g of seaweed [30]. However, Foley et al. [44] reported the use of 80% ethanol as an optimum solvent for the extraction of sulphated polysaccharides from *A. nodosum* at 70 °C for 12 h of extraction. Despite the potential applications of polyphenols from macroalgae, their widespread industrial applications have been hindered as these compounds are difficult to separate, purify and characterise [13]. Carbohydrates are normally co-extracted and tightly bound to phenols, carotenoids and phytosterols [13,45], and thus, these polysaccharides need to be eliminated to improve the purification processes of polyphenols. As previously mentioned, the low co-extraction of TSC in this study for all the novel technologies explored, with exception of HAE, may indicate that the use of 50% ethanol as a solvent of extraction combined with the technologies achieving high yields of polyphenols may be a promising strategy when targeting these compounds, reducing subsequent purification steps.

### 2.2. Effect of Novel Technologies on the Antioxidant Capacity of the Extracts

**Figure 2:**
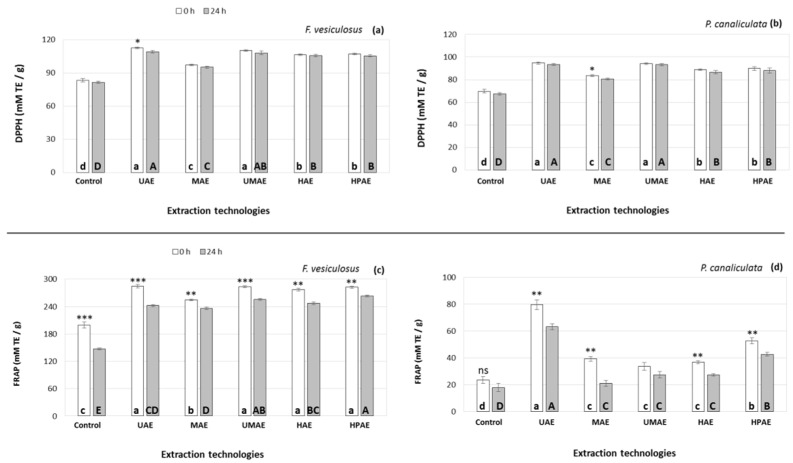
Antioxidant properties DPPH (**a**,**b**) and FRAP (**c**,**d**) of extracts obtained from *F. vesiculosus* and *P. canaliculata* by using novel extraction technologies and extraction times post-treatment (0 and 24 h) versus control treatment. Results are expressed as average ± standard deviation (*n* = 3). Different letters indicate statistical differences (*p* < 0.05) on the antioxidant properties of the macroalgal extracts between the different technological treatments incubated either at 0 h (lower case letters) or 24 h (upper case letters). Statistical differences between the extraction yields following different extraction times (0 and 24 h) within the same extraction treatment are reflected in the top figure as: * *p* < 0.05, ** *p* < 0.01 and *** *p* < 0.001.

The antioxidant properties (DPPH and FRAP) of the extracts obtained by using multiple extraction technologies and extraction times from *F. vesiculosus* and *P. canaliculata* are represented in Figure 2. There was a significant variation in the antioxidant properties of the extracts depending on the macroalgal species, novel technology and extraction time used. Overall, extracts from *F. vesiculosus* had higher DPPH (ranging from 95 to 112 mM trolox equivalents (TE)/g dried extract) and FRAP (236–285 mM TE/g) compared to those of *P. canaliculata,* ranging from 80–95 and 21–80 mM TE/g for DPPH and FRAP, respectively. Similarly to the phytochemicals analysed in this study, additional extraction of the samples for 24 h post-treatment did not improve the antioxidant properties of the extracts generated by any novel technology.

All the extraction technologies generated extracts with higher antioxidant activities (DPPH and FRAP) compared to the control group. Moreover, the different novel extraction technologies applied had a similar effect on the antioxidant properties of the extracts generated from both *F. vesiculosus* and *P. canaliculata*. The highest DPPH antioxidant activities were achieved in extracts generated by UAE and UMAE (ranging from 110 to 112 mM trolox equivalents (TE)/g in *F. vesiculosus* and 94–94 mM TE/g in *P. canaliculata*) followed by HAE and HPAE (106–107 mM TE/g in *F. vesiculosus* and 89–90 mM TE/g in *P. canaliculata*), and the lowest DPPH radical scavenging activities were achieved in the extracts obtained by MAE (97 ± 0.1 mM TE/g in *F. vesiculosus* and 83 ± 0.6 mM TE/g in *P. canaliculata*).

In the case of FRAP, the extracts obtained by different technological treatments did not behave in the same way in both macroalgae. In *F. vesiculosus*, the extracts generated by MAE had the lowest FRAP activities (255 ± 3 mM TE/g), with no differences in FRAP activity between the other extracts achieved by any the other novel technologies, ranging from 277 to 285 mM TE/g, despite the fact that the phytochemicals analysed in these samples significantly varied amongst the treatments. In the case of *P. canaliculata*, the extracts containing maximum FRAP activity were those generated by UAE (80 ± 3 mM TE/g), followed by HPAE (53 ± 2 mM TE/g), and the lowest FRAP activity (ranging from 34–39 mM TE/g) was achieved in extracts generated by MAE, UMAE and HAE without statistical differences amongst the treatments despite their different phytochemical composition.

**Figure 3:**
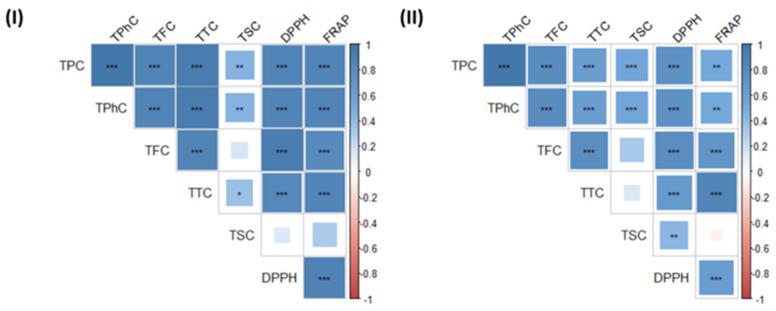
Correlation matrices of the composition and antioxidant properties in extracts obtained from (**I**) *F. vesiculosus* and (**II**) *P. canaliculata*. The sign of the correlations is colour coded (blue = + and red = –) and the strength of the correlations (0–1) relates to the size of each square and depth of each colour. Abbreviations in the figure are as follows: total phenolic content (TPC), total phlorotannin content (TPhC), total flavonoid content (TFC), total tannin content (TTC), total sugar content (TSC), 1,1-diphenyl-2-picryl-hydrazil radical scavenging activity (DPPH) and ferric reducing antioxidant power (FRAP). The statistical significance of the correlations is indicated in the figure as * *p* < 0.05, ** *p* < 0.01, *** *p* < 0.001.

To understand the relationship between the phytochemical composition of the extracts and their antioxidant properties in *F. vesiculosus* and *P. canaliculata*, correlation matrices were analysed in Figure 3. Both antioxidant activities (DPPH and FRAP) were positively and significantly correlated with each other in both macroalgal species, as well as with the levels of TPC, TPhC, TFC and TTC. In the case of *F. vesiculosus*, the antioxidant properties of the extracts were not correlated with TSC, and in *P. canaliculata* TSC was only correlated with the levels of DPPH. These results are in agreement with previous studies associating the polyphenol contents of macroalgae with high antioxidant properties [7,46,47,48]. Thereby, macroalgae react to environmental stressors by increasing the production of antioxidant phytochemicals to avoid any structural and metabolic changes due to the increased levels of oxidising agents (free radicals and other reactive species) [46]. Several studies have linked an increased production of TPC and antioxidant activities of macroalgae during spring and summer seasons as a defence mechanism against oxidative stress [7,47,48]. Thus, the amount of phytochemicals and associated antioxidant activity in macroalgae are strongly influenced by the environmental conditions and the seasons they are harvested. Moreover, studies extracting TPC, TPhC, TFC and TTC have also focused on the antioxidant activity of these compounds, as it is essential for the potential use of polyphenols for high value applications as pharmaceuticals, nutraceuticals and cosmeceuticals [5,6,49].

**Figure 4:**
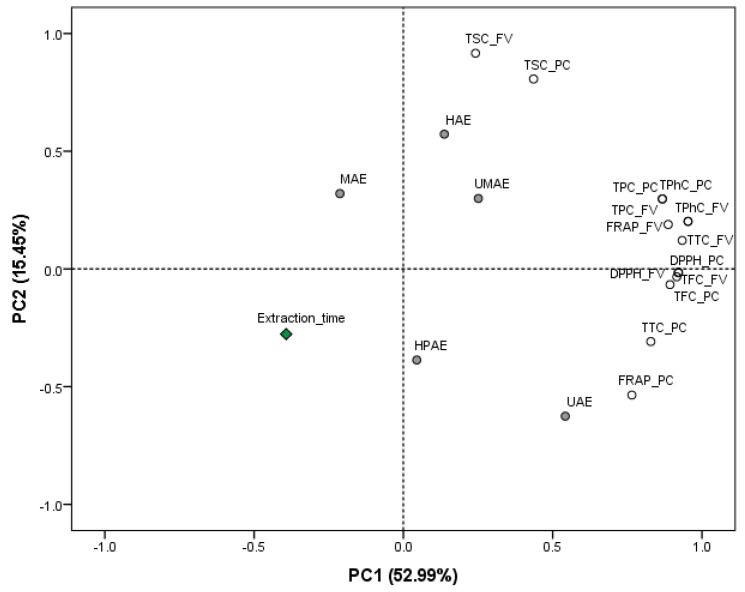
Principal component analysis (PCA) scatter plot representing the scores for the extraction technologies and extraction time applied for the extraction of phytochemicals (total phenolic content (TPC), total phlorotannin content (TPhC), total flavonoid content (TFC), total tannin content (TTC) and total sugar content (TSC)) and antioxidants (DPPH and FRAP) from macroalgae, FV (*F. vesiculosus*) and PC (*P. canaliculata*).

Principal component analysis (PCA) was also performed to obtain an overview of the similarities and differences in the phytochemical and antioxidant composition of the extracts produced from *F. vesiculosus* and *P. canaliculata* following different extraction technological treatments and extraction times (see Figure 4). The two principal components (PC) extracted from the data set explained a combined 68.44% of the variation of the data. PC1 explained 52.99% of this variation and clustered all phytochemical and antioxidant properties from both macroalgae together with the application of UAE in the right side of PC1. The second component explained the remaining variability of the data set and clearly separated the recovery of TSC from both macroalgae together with HAE treatment. These results agree with the previous literature, emphasising the efficiency of multiple HAE protocols for the extraction of carbohydrates from multiple macroalgal species [8,36,37]. Thus, the use of methods achieving high yields of phytochemicals (TPC, TPhC, TFC and TTC) and antioxidant properties, such as UAE combined with 50% ethanolic solutions, as indicated by the results of this study, may be the most promising strategy to target the extraction of polyphenols from *F. vesiculosus* and *P. canaliculata* while decreasing the co-extraction of undesirable carbohydrates, reducing the use of further purification strategies for the commercial exploitation of these compounds.

## 3. Materials and Methods

### 3.1. Macroalgal Biomass and Processing

Brown macroalgae *F. vesiculosus* and *P. canaliculata* were collected during November 2017 by Quality Sea Veg in Co Donegal (Ireland). The biomass was washed to remove epiphytes, sand and other debris, chopped and oven dried (55 °C, 5 days). Oven-dried samples were ground and sieved to a uniform particle size (1 mm), vacuum-packed and stored in a cool and dry place for further extraction experiments.

### 3.2. Extraction Procedures

The macroalgal biomass was mixed with ethanol solutions (50% *v/v*) at a biomass/solvent ratio of 1:10 *w/v*, and the mixtures were stirred for 10 min prior to the extraction processes. The mixtures were then subjected to novel extraction techniques including ultrasound-assisted extraction (UAE), microwave-assisted extraction (MAE), ultrasound–microwave-assisted extraction (UMAE), hydrothermal-assisted extraction (HAE) and high-pressure-assisted extraction (HPAE). All the extraction procedures were performed in duplicate and repeated twice (*n* = 4). UAE treatments were performed using a semi-industrial UIP500hdT 26 kHz (Hielscher Ultrasound Technology, Teltow, Germany) at 100% ultrasonic amplitude. MAE was performed in a microwave oven (Panasonic NN-CF778S0, Bracknell, UK; 2450 MHz) at 250 W. UMAE was performed by coupling the aforementioned devices, UIP500hdT 26 kHz and Panasonic NN-CF778S0 2450 MHz, keeping the ultrasonic power (100%) and microwave power (250 W) constant through the extraction process. HAE was performed using an autoclave (Tomy SS-325, Tomy Seiko, Tokyo, Japan) at 100 °C and 15 psi. HPAE was performed in the HPP Tolling facility (Dublin, Ireland) using a 200 L Hiperbaric HPP (Hiperbaric, Burgos, Spain) at 600 MPa. All the extraction procedures were performed at fixed extraction time (10 min), solvent (50% ethanol solution) and macroalgae:solvent ratio (1:10 *w/v*) based on previous optimisation protocols for the recovery of polyphenols from brown macroalgae [24]. Selection of 50% ethanol as an extraction solvent in this study was based upon previous findings wherein 50% ethanolic solution resulted the highest recovery of total phenols from macroalgae compared to 30% and 70% ethanol solutions [24]. Control samples were performed at room temperature using a magnetic stirring plate (IKA^®^ C-MAG HS 7, Staufen, Germany) for the same duration as the technological treatments (10 min).

After the process of 10 min extraction (fixed for each extraction method), the samples were either processed immediately (termed as—0 h extraction samples) or stored for an additional 24 h (termed as—24 h extraction samples) in closed containers under refrigeration at 4 °C. The objective of storing the extracts for 24 h after the extraction process was to evaluate if more phytochemicals leach out from treated algal cell wall or if they degrade, which would have an impact on total antioxidant activity of the extracts. After each extraction period (0 or 24 h), the samples were filtered through Whattman^®^ number 3 (GE Healthcare, Buckinghamshire, UK) and the ethanol of the extracts was evaporated under vacuum (Rotavapor R-100, Büchi Labortechnik AG, Flawil, Switzerland). The extracts were freeze-dried (FreeZone 6, Labconco Corporation, Kansas City, MO, USA) until constant dry weight (94.82 ± 0.14% dry weight). The extracts were then vacuum packed and stored at −20 °C for further analyses. The extraction yields for each treatment and extraction time were calculated using the formula: Extraction yield (mg/g) = weight of dry extract (mg)/weight of dry sample (g).

### 3.3. Phytochemical Analyses

All the phytochemical analyses of the extracts were performed in triplicate (*n* = 3). All the freeze-dried extracts were dissolved in ethanol, a stock concentration of 1 mg/mL was prepared and used in analyses. All standards used in this study were purchased from Sigma-Aldrich (Arklow, Co. Wicklow, Ireland).

#### 3.3.1. Total Phenolic Content (TPC) and Total Phlorotannin Content (TPhC) Analyses

TPC and TPhC were determined following the Folin–Ciocalteu reagent method as described by Rajauria et al. [50]. A total of 100 µL of sample/standard was mixed with 2 mL of sodium carbonate solution (Na_2_CO_3_ solution, 2% *w/v*). Following 2 min extraction, 100 µL of Folin–Ciocalteu’s solution (1 M) was added to all the mixtures and the reaction was incubated for 30 min at room temperature in dark conditions. The absorbance of the reactions was read at 720 nm in a spectrophotometer (UVmini-1240, Shimadzu, Kyoto, Japan). Distilled water (instead of extract or standard) along with other reagents was used as blank and gallic acid (>97.5% purity) and phloroglucinol (>99% purity) at concentrations of 25–300 mg/L were used as standards for TPC and TPhC, respectively. TPC results were expressed as mg GAE/g and TPhC as mg PGE/g.

#### 3.3.2. Total Flavonoid Content (TFC)

TFC was determined following the protocol described by Liu et al. [51] with slight modifications. Briefly, 250 μL of sample/standard was mixed with 1.475 mL of distilled water and 75 μL of sodium nitrite solution (NaNO_2_ solution 5% *w/v*) and the reaction was allowed to stand for 6 min. A total of 150 μL of aluminium chloride hexahydrate solution (AlCl_3_·6H_2_O solution 10% *w/v*) was added, mixed thoroughly and allowed to stand for 5 min. Following extraction, 0.5 mL of sodium hydroxide solution (NaOH solution 1 M) was added and the absorbance of the reactions was read at 510 nm in a spectrophotometer. Distilled water (instead of extract or standard) along with other reagents was used as blank and quercetin (>95% purity) was used as standard at concentrations of 30–150 mg/L. TFC results were expressed as mg QE/g.

#### 3.3.3. Total Sugar Content (TSC)

TSC was determined by the phenol–sulphuric acid method as described by [52] with minor modifications. Briefly, 100 μL of sample/standard was mixed with 100 μL of phenol solution (0.8% *w/v*), followed by 2 mL of concentrated sulphuric acid (H_2_SO_4_ 95–98%). The mixtures were allowed to stand for 10 min at room temperature and later incubated at 30 °C in a water bath for 20 min. The absorbance of the reaction was read in a spectrophotometer at 490 nm. Distilled water was used as blank and D-glucose (>99.5% purity) as a standard at concentrations of 50–250 mg/L. TSC results were expressed as mg GlcE/g.

#### 3.3.4. Total Tannin Content (TTC)

TTC was analysed following the protocol as described by Liu et al. [51]. A total of 50 μL of sample was mixed with 1.5 mL of a methanolic solution of vanillin (4% *w/v*) (99% purity, Sigma-Aldrich, Arklow, Co. Wicklow, Ireland) and 750 μL of hydrochloric acid (HCl 37% *w/v*). The solutions were mixed thoroughly and incubated in dark conditions at room temperature for 20 min. The absorbance of the reaction was read in a spectrophotometer at 500 nm. Distilled water was used as blank and (+)-catechin hydrate (>98% purity) was used as standard at concentrations of 15–150 mg/L. TTC results were expressed as mg ChE/g.

### 3.4. Antioxidant Analyses

All the antioxidant analyses of the extracts were performed in triplicate (*n* = 3).

#### 3.4.1. 1,1-Diphenyl-2-Picryl-Hydrazil (DPPH) Radical Scavenging Activity

DPPH assay was performed following the protocol as described by Sridhar and Charles [53]. A total of 700 μL of a methanolic DPPH solution (100 μM) was added to 700 μL of sample/standard, incubating the mixtures at room temperature in the dark for 20 min. The absorbance of the reactions was measured against blank (methanol without DPPH solution) at a wavelength of 515 nm in a spectrophotometer. Trolox (97% purity) was used as standard at concentrations ranging from 10–150 mM/L. DPPH results were expressed as mM TE/g.

#### 3.4.2. Ferric Reducing Antioxidant Power (FRAP) Assay

FRAP assay was performed following the method described by Benzie and Strain [54]. Fresh FRAP working solutions were prepared by mixing 300 mM acetate buffer, pH 3.6; 10 mM 2,4,6-Tri(2-pyridyl)-s-triazine in 40 mM hydrochloric acid and 20 mM Iron(III) chloride hexahydrate in the ratio of 10:1:1, *v/v*/*v*. A total of 2.5 mL of pre-heated FRAP working solution (37 °C) was added to 83 μL of sample/standard and the mixtures were incubated 10 min in dark conditions at room temperature. The absorbance of the reactions was measured at 593 nm in a spectrophotometer against a reagent blank (FRAP working solution). Trolox was used as standard at concentrations of 10–150 mM/L. FRAP results were expressed as mM TE/g.

### 3.5. Statistical Analyses

The influences of the macroalgal species, extraction technology and extraction time post-treatment on the extracted phytochemicals (TPC, TPhC, TFC, TSC and TTC) and antioxidant activities (DPPH and FRAP) were analysed by multivariate general linear model in SPSS version 24.0. The differences were further analysed by either Tukey’s HSD post hoc tests or Student’s *t*-test. Differences were considered to be significant at *p*-values < 0.05. The relationships between the composition of the extracts and their antioxidant properties were explored in R version 4.0.2 [55]. The statistical packages “ggplot2” and “corrplot” were used to generate the correlation matrix [56] and “cor.mtest” produced the *p*-values for the Pearson’s correlation matrix. The variance in the data set was further analysed by principal component analysis (PCA) using Equamax rotation with Kaiser normalisation extracting the weight for each component with eigenvalues higher than 1 using SPSS version 24.0.

## 4. Conclusions

The yields of compounds extracted from *F. vesiculosus* (173.33 to 354.67 mg dried extract per g macroalgae) were higher when compared to *P. canaliculata* (147.00 to 240.33 mg dried extract/g macroalgae). The yields were influenced by the technology used and, overall, the highest were achieved using UMAE (354.67 ± 2.33 for *F. vesiculosus* and 240.33 ± 1.20 for *P. canaliculata*), followed by UAE (316.33 ± 4.63 and 214.33 ± 2.60 for *F. vesiculosus* and *P. canaliculata*, respectively). Following the extraction treatment, additional extraction (24 h) did not improve the yields of phytochemicals or the antioxidant properties of the extracts, indicating possible precipitation or degradation loss of the compounds dissolved for prolonged periods of time in ethanolic solutions. When analysing the extraction of phytochemicals using multiple novel technologies without additional extraction after extraction, overall UAE achieved the highest yields of most phytochemicals from both *F. vesiculosus* (445.0 ± 4.6 mg GAE/g, 362.9 ± 3.7 mg PGE/g, 286.3 ± 7.8 mg QE/g, and 189.1 ± 4.4 mg ChE/g) and *P. canaliculata* (250.6 ± 6 mg GAE/g, 203.9 ± 4.9 mg PGE/g, 122.6 ± 3.4 mg QE/g and 79.5 ± 4.6 mg ChE/g). Moreover, UMAE also achieved high yields of TPC, TPhC and TFC from *P. canaliculata*. Overall, the extraction procedures using 50% ethanol as solvent achieved low TSC yields, except when using HAE, from both macroalgae. In the case of the antioxidant properties, the extracts with the highest DPPH radical scavenging activities were extracted by UAE and UMAE and the lowest by MAE from both *F. vesiculosus* and *P. canaliculata*. Meanwhile, strong variations were appreciated depending on the extraction technology and macroalgal species considered in the case of FRAP activity. Both DPPH and FRAP were positively correlated with the levels of TPC, TPhC, TFC and TTC in both macroalgal species. This study evaluated the effectiveness of multiple extraction technologies for the recovery of phytochemicals (keeping a fixed solvent type, concentration and extraction time) from macroalgae. Further, studies are needed optimising the use of novel extraction technologies, varying extraction time and the polarity of the extraction solvents targeting multiple compounds from macroalgae in order to establish efficient extraction protocols to allow the future commercialisation of these compounds. Moreover, further chemical characterisation of the extracted compounds as well as confirmation of their biological effects in vivo will also be needed in order to commercialise these compounds. Based on the results of this study, the use of UAE combined with 50% ethanolic solution as an extraction solvent could be a promising strategy targeting the extraction of TPC, TPhC, TFC and TTC, while reducing the co-extraction of undesirable carbohydrates from both *F. vesiculosus* and *P. canaliculata*, with promising applications when using these compounds as pharmaceuticals, nutraceuticals and cosmeceuticals.

## Figures and Tables

**Table 1:** Effect of novel extraction technologies and extraction time post-treatment (0 and 24 h) on total phenolic content (TPC), total phlorotannin content (TPhC), total flavonoid content (TFC), total tannin content (TTC) and total sugar content (TSC) extracted from *F. vesiculosus* and *P. canaliculata*.

Macroalgae sp.	Extraction Technologies	TPC (mg GAE/g)		TPhC (mg PGE/g)		TFC (mg QE/g)		TTC (mg ChE/g)		TSC (mg GlcE/g)	
		0 h	24 h	0 h	24 h	0 h	24 h	0 h	24 h	0 h	24 h
*F. vesiculosus*	Control	316.4 ± 3.9 d	293.2 ± 5.9 e **	257.7 ± 3.2 d	238.7 ± 4.9 e **	180.4 ± 7.8 e	129.3 ± 6.4 e **	123.2 ± 3.4 d	91.3 ± 3.8 c ***	136.1 ± 9.1 c	116.6 ± 8.9 b ^ns^
	UAE	445.0 ± 4.6 a	413.4 ± 5.1 a **	362.9 ± 3.7 a	337.1 ± 4.2 a **	286.3 ± 7.8 a	285.6 ± 7.7 a ^ns^	189.1 ± 4.4 a	168.4 ± 7.1 a *	130.7 ± 6.2 c	111.2 ± 9.6 b *
	MAE	391.2 ± 6.0 c	375.9 ± 5.3 c *	318.9 ± 4.9 c	306.4 ± 4.4 c *	202.6 ± 3.4 d	198.1 ± 7.1 d ^ns^	161.0 ± 5.6 bc	142.4 ± 4.4 b *	199.9 ± 9.2 b	162.7 ± 7.9 a **
	UMAE	431.2 ± 4.5 b	392.2 ± 7.5 b **	351.6 ± 3.7 b	319.7 ± 6.1 b **	268.5 ± 6.4 b	266.3 ± 1.3 b *	172.8 ± 7.8 b	151.3 ± 4.4 b **	194.5 ± 9.6 b	157.6 ± 6.0 a ^ns^
	HAE	433.2 ± 6.2 ab	375.5 ± 2.1 c ***	353.3 ± 5.1 ab	306.1 ± 1.7 c ***	253.0 ± 5.6 b	250.0 ± 4.4 b ^ns^	166.9 ± 6.7 b	146.9 ± 2.2 b **	239.4 ± 2.9 a	164.4 ± 5.0 a ***
	HPAE	387.9 ± 3.8 c	356.1 ± 2.4 d ***	316.2 ± 3.1 c	290.2 ± 2.0 d ***	231.5 ± 1.3 c	228.5 ± 7.1 c ^ns^	148.4 ± 3.4 c	138.0 ± 9.7 b ^ns^	123.7 ± 5.6 c	81.9 ± 2.9 c ***
*P. canaliculata*	Control	174.8 ± 5.5 d	158.3 ± 7.8 e *	141.9 ± 4.5 d	128.3 ± 6.4 e *	82.6 ± 8.4 b	80.4 ± 6.8 c ^ns^	33.6 ± 2.2 d	32.1 ± 1.3 d ^ns^	35.7 ± 5.0 d	58.4 ± 7.2 c **
	UAE	250.6 ± 6.0 a	182.3 ± 7.8 d ***	203.9 ± 4.9 a	148.0 ± 6.4 d ***	122.6 ± 3.4 a	108.5 ± 5.6 a *	79.5 ± 4.6 a	62.4 ± 2.2 a **	59.1 ± 6.1 c	42.4 ± 8.7 d ^ns^
	MAE	205.3 ± 3.6 c	192.6 ± 7.1 bd ^ns^	166.8 ± 2.9 c	156.4 ± 5.8 bd ^ns^	93.7 ± 7.8 b	84.1 ± 6.4 c ^ns^	35.8 ± 3.8 cd	32.8 ± 2.6 cd ^ns^	123.1 ± 6.1 b	45.1 ± 8.1 d ***
	UMAE	238.4 ± 3.0 ab	210.4 ± 3.4 a ***	193.9 ± 2.5 ab	171.0 ± 2.8 a ***	113.7 ± 3.4 a	102.6 ± 6.8 ab ^ns^	47.6 ± 4.6 b	41.7 ± 3.4 b *	109.1 ± 3.1 b	92.4 ± 7.2 b ^ns^
	HAE	236.2 ± 1.9 b	202.3 ± 3.2 abc ***	192.1 ± 1.6 b	164.4 ± 2.7 abc ***	113.0 ± 4.6 a	87.8 ± 6.7 bc **	55.0 ± 2.6 b	41.0 ± 4.6 bc *	175.7 ± 9.0 a	123.1 ± 7.0 a **
	HPAE	226.5 ± 5.7 b	194.1 ± 3.2 cd **	184.2 ± 4.7 b	157.7 ± 2.6 cd **	110.7 ± 5.6 a	93.0 ± 6.8 abc *	46.9 ± 5.9 bc	37.3 ± 3.4 bcd ^ns^	25.1 ± 3.1 d	15.7 ± 1.2 d *

Results are expressed as average ± standard deviation (*n* = 3). The units of the phytochemical compounds analysed are expressed as follows: TPC (mg gallic acid equivalents (GAE)/g dried weight extract), TPhC (mg phloroglucinol equivalents (PGE)/g dried weight extract), TFC (mg quercetin equivalents (QE)/g dried weight extract), TTC (mg catechin equivalents (ChE)/g dried weight extract) and TSC (mg glucose equivalents (GlcE)/g dried weight extract). Different letters indicate statistical differences (*p* < 0.05) on the recovery of compounds at extraction times post-treatment 0 or 24 h for each individual macroalgal species. Differences between extraction times 0 and 24 h within the same technological treatment are represented in superscripts in the table: ^ns^ (not-significant), * (*p* < 0.05), ** (*p* < 0.01) and *** (*p* < 0.001).

## Data Availability

Not applicable as all the data is contained in the manuscript.

## References

[1] Guiry M., Guiry G., AlgaeBase World-Wide Electronic Publication. www.algaebase.org.

[2] Chojnacka K., Saeid A., Witkowska Z., Tuhy L. (2012). Biologically active compounds in seaweed extracts-the prospects for the application. Open Conf. Proc. J..

[3] Fatima F., Løvstad H.S., Rohan S., Pedro M., Zhengyong Y. (2018). The Global Statusof Seaweed Production, Trade and Utilization.

[4] Zhu X., Healy L., Zhang Z., Maguire J., Sun D., Tiwari B.K. (2021). Novel postharvest processing strategies for value-added applications of marine algae. J. Sci. Food Agric..

[5] Cotas J., Leandro A., Monteiro P., Pacheco D., Figueirinha A., Gonçalves A.M.M., Da Silva G.J., Pereira L. (2020). Seaweed phenolics: From extraction to applications. Mar. Drugs.

[6] Mekinić I.G., Skroza D., Šimat V., Hamed I., Čagalj M., Perković Z.P. (2019). Phenolic content of brown algae (pheophyceae) species: Extraction, identification, and quantification. Biomolecules.

[7] Garcia-Vaquero M., Rajauria G., Miranda M., Sweeney T., Lopez-Alonso M., O’Doherty J. (2021). Seasonal variation of the proximate composition, mineral content, fatty acid profiles and other phytochemical constituents of selected brown macroalgae. Mar. Drugs.

[8] Rajauria G., Jaiswal A.K., Abu-Ghannam N., Gupta S. (2010). Effect of hydrothermal processing on colour, antioxidant and free radical scavenging capacities of edible Irish brown seaweeds. Int. J. Food Sci. Technol..

[9] Qin Y., Qin Y. (2018). Health benefits of bioactive seaweed substances. Bioactive Seaweeds for Food Applications: Natural Ingredients for Healthy Diets.

[10] Tierney M.S., Smyth T.J., Hayes M., Soler-Vila A., Croft A.K., Brunton N. (2013). Influence of pressurised liquid extraction and solid–liquid extraction methods on the phenolic content and antioxidant activities of I rish macroalgae. Int. J. Food Sci. Technol..

[11] Chater P.I., Wilcox M., Cherry P., Herford A., Mustar S., Wheater H., Brownlee I., Seal C., Pearson J. (2016). Inhibitory activity of extracts of Hebridean brown seaweeds on lipase activity. Environ. Boil. Fishes.

[12] Yuan Y., Zhang J., Fan J., Clark J., Shen P., Li Y., Zhang C. (2018). Microwave assisted extraction of phenolic compounds from four economic brown macroalgae species and evaluation of their antioxidant activities and inhibitory effects on α-amylase, α-glucosidase, pancreatic lipase and tyrosinase. Food Res. Int..

[13] Tierney M.S., Smyth T.J., Rai D.K., Soler-Vila A., Croft A.K., Brunton N. (2013). Enrichment of polyphenol contents and antioxidant activities of Irish brown macroalgae using food-friendly techniques based on polarity and molecular size. Food Chem..

[14] Garcia-Vaquero M., Ummat V., Tiwari B., Rajauria G. (2020). Exploring ultrasound, microwave and ultrasound-microwave assisted extraction technologies to increase the extraction of bioactive compounds and antioxidants from brown macroalgae. Mar. Drugs.

[15] Getachew A.T., Jacobsen C., Holdt S.L. (2020). Emerging technologies for the extraction of marine phenolics: Opportunities and challenges. Mar. Drugs.

[16] Tiwari B.K. (2015). Ultrasound: A clean, green extraction technology. Trends Anal. Chem..

[17] Obluchinskaya E.D., Daurtseva A.V., Pozharitskaya O.N., Flisyuk E.V., Shikov A.N. (2019). Natural deep eutectic solvents as alternatives for extracting phlorotannins from brown algae. Pharm. Chem. J..

[18] Anastas P.T., Warner J.C. (2000). Green Chemistry: Theory and Practice.

[19] Kadam S.U., Tiwari B.K., O’Donnell C. (2013). Application of novel extraction technologies for bioactives from marine algae. J. Agric. Food Chem..

[20] Li Y., Fu X., Duan D., Liu X., Xu J., Gao X. (2017). Extraction and identification of phlorotannins from the brown alga, sargassum fusiforme (Harvey) setchell. Mar. Drugs.

[21] Montero L., Sánchez-Camargo A.D.P., García-Cañas V., Tanniou A., Stiger-Pouvreau V., Russo M., Rastrelli L., Cifuentes A., Herrero M., Ibáñez E. (2016). Anti-proliferative activity and chemical characterization by comprehensive two-dimensional liquid chromatography coupled to mass spectrometry of phlorotannins from the brown macroalga Sargassum muticum collected on North-Atlantic coasts. J. Chromatogr. A.

[22] Puspita M., Déniel M., Widowati I., Radjasa O.K., Douzenel P., Marty C., Vandanjon L., Bedoux G., Bourgougnon N. (2017). Total phenolic content and biological activities of enzymatic extracts from Sargassum muticum (Yendo) Fensholt. Environ. Boil. Fishes.

[23] Hassan I.H., Pham H.N.T., Nguyen T.H. (2021). Optimization of ultrasound-assisted extraction conditions for phenolics, antioxidant, and tyrosinase inhibitory activities of Vietnamese brown seaweed (Padina australis). J. Food Process. Preserv..

[24] Ummat V., Tiwari B.K., Jaiswal A.K., Condon K., Garcia-Vaquero M., O’Doherty J., O’Donnell C., Rajauria G. (2020). Optimisation of ultrasound frequency, extraction time and solvent for the recovery of polyphenols, phlorotannins and associated antioxidant activity from brown seaweeds. Mar. Drugs.

[25] Magnusson M., Yuen A.K., Zhang R., Wright J.T., Taylor R.B., Maschmeyer T., De Nys R. (2017). A comparative assessment of microwave assisted (MAE) and conventional solid-liquid (SLE) techniques for the extraction of phloroglucinol from brown seaweed. Algal Res..

[26] Harnedy P., FitzGerald R.J. (2013). Extraction of protein from the macroalga Palmaria palmata. LWT Food Sci. Technol..

[27] Mabeau S., Kloareg B. (1987). Isolation and analysis of the cell walls of brown algae: *Fucus spiralis*, *F. ceranoides*, *F. vesiculosus*, *F. serratus*, *Bifurcaria bifurcata* and *Laminaria digitata*. J. Exp. Bot..

[28] Deniaud-Bouët E., Kervarec N., Michel G., Tonon T., Kloareg B., Hervé C. (2014). Chemical and enzymatic fractionation of cell walls from Fucales: Insights into the structure of the extracellular matrix of brown algae. Ann. Bot..

[29] Tai Y., Shen J., Luo Y., Qu H., Gong X. (2020). Research progress on the ethanol precipitation process of traditional Chinese medicine. Chin. Med..

[30] Vázquez-Rodríguez B., Gutiérrez-Uribe J.A., Antunes-Ricardo M., Santos-Zea L., Cruz-Suárez L.E. (2020). Ultrasound-assisted extraction of phlorotannins and polysaccharides from *Silvetia compressa* (Phaeophyceae). J. Appl. Phycol..

[31] Deniaud-Bouët E., Hardouin K., Potin P., Kloareg B., Hervé C. (2017). A review about brown algal cell walls and fucose-containing sulfated polysaccharides: Cell wall context, biomedical properties and key research challenges. Carbohydr. Polym..

[32] Amarante S.J., Catarino M.D., Marçal C., Silva A.M.S., Ferreira R., Cardoso S.M. (2020). Microwave-assisted extraction of phlorotannins from fucus vesiculosus. Mar. Drugs.

[33] Wen L., Zhang Z., Zhao M., Senthamaraikannan R., Padamati R.B., Sun D., Tiwari B.K. (2019). Green extraction of soluble dietary fibre from coffee silverskin: Impact of ultrasound/microwave-assisted extraction. Int. J. Food Sci. Technol..

[34] Yang J.-S., Mu T.-H., Ma M.-M. (2019). Optimization of ultrasound-microwave assisted acid extraction of pectin from potato pulp by response surface methodology and its characterization. Food Chem..

[35] Lianfu Z., Zelong L. (2008). Optimization and comparison of ultrasound/microwave assisted extraction (UMAE) and ultrasonic assisted extraction (UAE) of lycopene from tomatoes. Ultrason. Sonochemistry.

[36] Garcia-Vaquero M., O’Doherty J.V., Tiwari B.K., Sweeney T., Rajauria G. (2019). Enhancing the extraction of polysaccharides and antioxidants from macroalgae using sequential hydrothermal-assisted extraction followed by ultrasound and thermal technologies. Mar. Drugs.

[37] Lemus A., Bird K., Kapraun D.F., Koehn F. (1991). Agar yield, quality and standing crop biomass of Gelidium serrulatum, Gelidium floridanum and Pterocladia capillacea in Venezuela. Food Hydrocoll..

[38] Lee W.-K., Namasivayam P., Ho C.-L. (2013). Effects of sulfate starvation on agar polysaccharides of Gracilaria species (Gracilariaceae, Rhodophyta) from Morib, Malaysia. Environ. Boil. Fishes.

[39] Mulchandani K., Kar J.R., Singhal R.S. (2015). Extraction of lipids from chlorella saccharophila using high-pressure homogenization followed by three phase partitioning. Appl. Biochem. Biotechnol..

[40] Li G.-Y., Luo Z.-C., Yuan F., Yu X.-B. (2017). Combined process of high-pressure homogenization and hydrothermal extraction for the extraction of fucoidan with good antioxidant properties from Nemacystus decipients. Food Bioprod. Process..

[41] Graiff A., Ruth W., Kragl U., Karsten U. (2015). Chemical characterization and quantification of the brown algal storage compound laminarin—A new methodological approach. Environ. Boil. Fishes.

[42] Michalak I., Chojnacka K. (2014). Algal extracts: Technology and advances. Eng. Life Sci..

[43] Zhang H., Birch J., Xie C., Yang H., Dias G., Kong L., Bekhit A.E.-D. (2018). Optimization of extraction parameters of antioxidant activity of extracts from New Zealand and Chinese Asparagus officinalis L root cultivars. Ind. Crop. Prod..

[44] Foley S.A., Szegezdi E., Mulloy B., Samali A., Tuohy M.G. (2012). Correction to an unfractionated fucoidan from ascophyllum nodosum: Extraction, characterization, and apoptotic effects in vitro. J. Nat. Prod..

[45] Calixto F.D.S. (2011). Dietary fiber as a carrier of dietary antioxidants: An essential physiological function. J. Agric. Food Chem..

[46] Dang T.T., Bowyer M.C., Van Altena I.A., Scarlett C.J. (2017). Comparison of chemical profile and antioxidant properties of the brown algae. Int. J. Food Sci. Technol..

[47] Parys S., Kehraus S., Pete R., Küpper F.C., Glombitza K.-W., König G.M. (2009). Seasonal variation of polyphenolics in *Ascophyllum* nodosum (Phaeophyceae). Eur. J. Phycol..

[48] Schiener P., Black K.D., Stanley M.S., Green D. (2015). The seasonal variation in the chemical composition of the kelp species Laminaria digitata, Laminaria hyperborea, Saccharina latissima and Alaria esculenta. Environ. Boil. Fishes.

[49] Fraga-Corral M., García-Oliveira P., Pereira A.G., Lourenço-Lopes C., Jimenez-Lopez C., Prieto M.A., Simal-Gandara J. (2020). Technological application of tannin-based extracts. Molecules.

[50] Rajauria G., Jaiswal A.K., Abu-Ghannam N., Gupta S. (2013). Antimicrobial, antioxidant and free radical-scavenging capacity of brown seaweed Himanthalia elongata from western coast of Ireland. J. Food Biochem..

[51] Liu S.-C., Lin J.-T., Wang C.-K., Chen H.-Y., Yang D.-J. (2009). Antioxidant properties of various solvent extracts from lychee (Litchi chinenesis Sonn.) flowers. Food Chem..

[52] Brummer Y., Cui S.W. (2005). Understanding carbohydrate analysis. Food Carbohydrates: Chemistry, Physical Properties and Applications.

[53] Sridhar K., Charles A.L. (2019). In vitro antioxidant activity of Kyoho grape extracts in DPPH and ABTS assays: Estimation methods for EC50 using advanced statistical programs. Food Chem..

[54] Benzie I.F.F., Strain J.J. (1996). The ferric reducing ability of plasma (FRAP) as a measure of “antioxidant power”: The FRAP assay. Anal. Biochem..

[55] R Core Team (2020). R: A Language and Environment for Statistical Computing.

[56] Friendly M. (2002). Corrgrams: Exploratory displays for correlation matrices. Am. Stat..

